# Early-Onset Alzheimer’s Disease: What Is Missing in Research?

**DOI:** 10.1007/s11910-020-01090-y

**Published:** 2021-01-19

**Authors:** Temitope Ayodele, Ekaterina Rogaeva, Jiji T. Kurup, Gary Beecham, Christiane Reitz

**Affiliations:** 1 The Taub Institute for Research on Alzheimer’s Disease and the Aging Brain, Columbia University, New York, NY USA; 2 The Gertrude H. Sergievsky Center, Columbia University, New York, NY USA; 3 Department of Neurology, Columbia University, New York, NY USA; 4 Tanz Centre for Research in Neurodegenerative Disease, University of Toronto, 60 Leonard Avenue, Toronto, ON M5T 0S8 Canada; 5 The John P. Hussman Institute for Human Genomics, University of Miami, Miami, FL USA; 6 Department of Epidemiology, Sergievsky Center, Taub Institute for Research on the Aging Brain, Columbia University, 630 W 168th Street, New York, NY 10032 USA

**Keywords:** Early-onset Alzheimer’s disease, Epidemiology, Genetics, Biomarkers, Neuropathology

## Abstract

**Purpose of Review:**

Early-onset Alzheimer’s disease (EOAD), defined as Alzheimer’s disease (AD) occurring before age 65, is significantly less well studied than the late-onset form (LOAD) despite EOAD often presenting with a more aggressive disease progression. The aim of this review is to summarize the current understanding of the etiology of EOAD, their translation into clinical practice, and to suggest steps to be taken to move our understanding forward.

**Recent Findings:**

EOAD cases make up 5–10% of AD cases but only 10–15% of these cases show known mutations in the *APP*, *PSEN1*, and *PSEN2*, which are linked to EOAD. New data suggests that these unexplained cases following a non-Mendelian pattern of inheritance is potentially caused by a mix of common and newly discovered rare variants. However, only a fraction of this genetic variation has been identified to date leaving the molecular mechanisms underlying this type of AD and their association with clinical, biomarker, and neuropathological changes unclear.

**Summary:**

While great advancements have been made in characterizing EOAD, much work is needed to disentangle the molecular mechanisms underlying this type of AD and to identify putative targets for more precise disease screening, diagnosis, prevention, and treatment.

## Introduction

Dementia is a term that is used to describe a category of diseases characterized by a decline in cognitive function that impairs daily function. To date, there are 50 million people that are living with dementia worldwide [1]. Alzheimer’s disease (AD), the most common form of dementia, accounts for 50–75% of dementia cases [2]. Age is considered to be a principal risk factor for AD, and it is used as a categorical marker [3, 4]. The two main disease subcategories include early onset (EOAD) and late onset (LOAD) of AD, based on an arbitrary cut-off set at the age when individuals start to present symptoms, typically 65 years old [3, 4]. However, a cut-off of 60 years is sometimes used reflecting the arbitrary nature underlying this categorization [5]. Among reported AD cases, EOAD accounts for 5–10% [6]. LOAD has a prevalence of ~ 3.9% worldwide and an estimated annual incidence of ~ 7.5 per 1000 individuals above the age of 60 [7]. In comparison, data on prevalence and incidence of EOAD are much more limited. The studies that have assessed EOAD show that the annual prevalence and incidence of people between the age of 45 and 64 are approximately 24.2/100,000 and 6.3/100,000, respectively [8, 9].

## Clinical Manifestations of EOAD

In general, the clinical manifestation of AD is characterized by a predominant impairment of anterograde episodic memory. This symptom is typically accompanied by a multitude of cognitive impairments in domains, such as visuospatial, language, and executive function [7]. The combination of the aforementioned characteristics contributes to a global cognitive decline, eventually leading to a total dependent state, and death [10]. Although this typical clinical presentation of memory-predominant phenotypes overlaps between LOAD and EOAD cases, a subset of EOAD cases show an atypical presentation of preserved episodic memory function but focal cortical symptoms relating to language, visuospatial, or executive function [11]. In 25% of EOAD cases, there is a distinct phenotype of non-memory symptoms, in particular apraxia, visual dysfunction, fluent or non-fluent aphasia, executive dysfunction, or dyscalculia, that is seen as the disease progresses [11–13]. In addition, individuals with EOAD often present with a more aggressive disease progression and a shorter relative survival time [12], with the rate of progression driven at least in part by the nature of the underlying causative variant (see below). EOAD cases have a greater pathological burden (neuritic plaques and neurofibrillary tangles) compared to LOAD [14].

There have been no systematic large-scale studies assessing to what extent the different thresholds used affect estimates of prevalence, incidence, or clinical, genetic, and neuropathological variability. There is significant variability in age of onset both within and across families, which is partly explained by known genetic, environmental, or stochastic factors [15]. While some autosomal dominant cases develop first symptoms as early as their late 20s, others develop the disease in their early 60s (prevalence increases with age) [8, 9]. Finally, in a recent study of mutation carriers and non-carrier in the Dominantly Inherited Alzheimer Network (DIAN) cohort, an observational study of families with *PSEN1*, *PSEN2* or *APP* causal AD mutations, the personality traits of neuroticism, and conscientiousness were correlated with years to symptom onset, markers of tau pathology in the cerebrospinal fluid (CSF), and longitudinal rates of cognitive decline [16]. This suggests that there is clinically significant variability that needs to be thoroughly investigated in order to better characterize the disease.

## Neuropathological Changes

The neuropathology of AD brains is characterized by extracellular accumulation of amyloid plaques, consisting of Aβ40 and Aβ42 peptides generated by the cleavage of APP, and intra-neuronal deposition of neurofibrillary tangles (NTF) composed of hyperphosphorylated tau protein (p-tau) [17]. These pathological hallmarks are often complemented by additional morphological changes including synaptic loss [18], neuronal loss [19], microglial activation [20], reactive astrocytes [21], neurovascular dysfunction [22], disruption of the blood brain barrier [23], and brain atrophy [24]. Up to 50% of AD patients exhibit concurrent α-synuclein (αSyn) pathology [25].

Both Aβ and NFT neuropathological changes in both EOAD and LOAD progressively involve brain regions and neuronal cell types following a characteristic pattern [26]. Aβ accumulates initially across neocortical regions, then the limbic system, diencephalon, basal forebrain, and lastly the cerebellum. In contrast, tau involves first the entorhinal cortex and hippocampus, resulting in early-stage neurofibrillary degeneration, synaptic and neuronal loss and regional atrophy, and subsequently (early/mid stage) the locus coeruleus, basal forebrain, and associated regions of the neocortex, followed by the primary sensory cortex.

The extent to which the neuropathology underlying EOAD differs from that of LOAD remains unclear. Some distinctions have been reported. Temporoparietal-precuneus atrophy is seen in both EOAD and LOAD cases, but EOAD cases are seen to have a higher burden of neuritic plaques and NFT in these regions as well as the frontal cortex (albeit to a lesser degree) when compared to LOAD patients [26]. The neocortex, particularly parietal and occipitoparietal regions, seems to experience a similar burden, while the hippocampus is more likely to be spared in EOAD [27, 28]. Finally, individuals who develop AD at a younger age more often than not seem to have “purer” AD pathology with less concomitant neuropathological changes than older AD patients who often show a plethora of pathologies besides inclusions typical for AD (i.e., Lewy bodies, TDP-43, vascular pathology) [29–31]. It is important to recognize that many of the neuropathological studies conducted have accounted for age of onset and/or cognitive function but not duration of disease.

There are current research efforts to synchronize and fill the gap of understanding of the neuropathological overlap between LOAD and autosomal dominant EOAD by implementing a uniform neuropathological assessment protocol [32]. While this effort will be highly valuable, systematic harmonization of brain tissue assessment from non-Mendelian EOAD cases accounting for the majority of EOAD cases [33] in a similar manner is lacking.

## Genetics of EOAD

While the late-onset form of AD has a heritability of between 70 and 80% [3, 34], the heritability of EOAD is higher amounting to 92–100% [3]. This correlates with the observation that up to 60% of EOAD subjects have at least one affected first-degree relative [33, 35].

### Mendelian EOAD

As described above, families affected by EOAD can either follow a Mendelian (M-EOAD) or non-Mendelian inheritance pattern (NM-EOAD). Mendelian EOAD is caused by variants in *APP* [36], *PSEN1* [37], or *PSEN2* [37, 38]. Known to date are close to 330 mutations (duplications and missense) in these three genes accounting for 10–15% (*APP*), 30–70% (*PSEN1*), and < 5% (*PSEN2*) of M-EOAD cases, respectively (https://www.alzforum.org/alzgene). While identification of these genetic variations has largely enhanced our understanding of AD pathogenesis, they only explain 10–15% of familial EOAD cases, leaving the vast proportion of cases unexplained [33, 39].

**Fig. 1:**
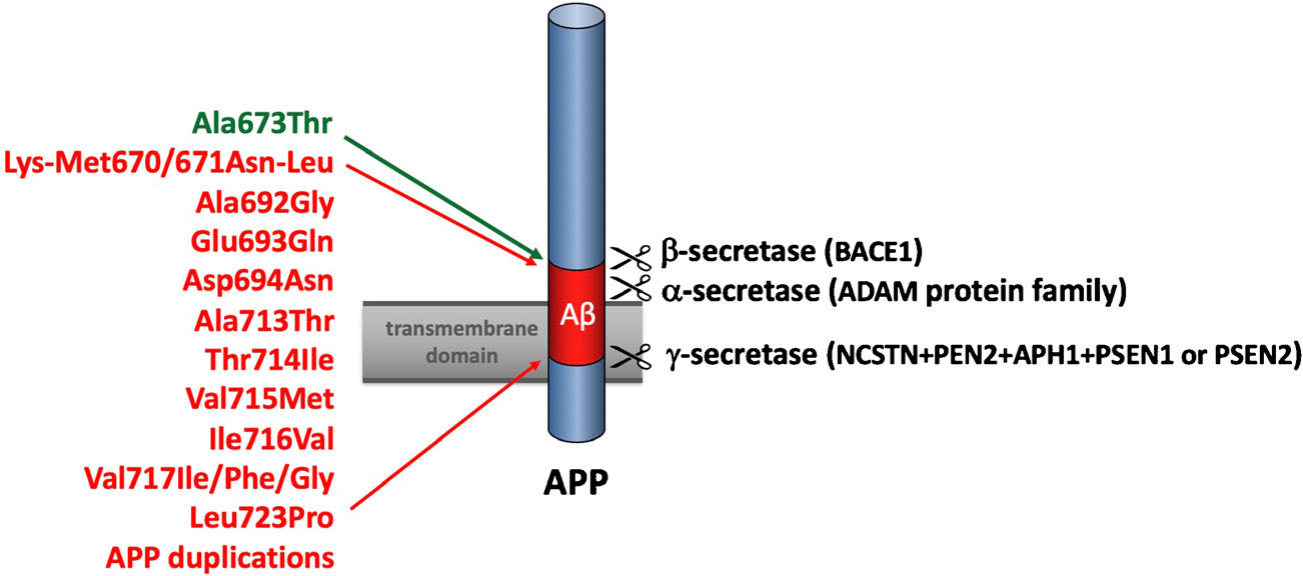
Representative APP mutations causing early-onset Alzheimer’s disease with or without hemorrhage or stroke (in red). All known pathogenic missense substitutions in APP are localized in or around the Aβ-domain encoded by exons 16 and 17. The protective APP variant (Ala673Thr) is indicated in green

Mutations in all three genes affect the amyloidogenic pathway leading to increased generation and aggregation of Aβ [40, 41]. Aβ peptides result from the cleavage of APP by β- and γ-secretases; PSEN1 and PSEN2 are components of the γ-secretase complex [42]. While most mutations in *APP* result in increased levels of Aβ aggregation and production [43], a protective *APP* variant (Icelandic mutation Ala673Thr) decreases Aβ levels by 40% [44] (Fig. 1). Of note, mutations in the same codon (Ala673Val) cause AD when inherited in a recessive fashion [45]. *APP* carriers tend to have a notable level of amyloid angiopathy in their brain, which can lead to cerebral hemorrhage and stroke (i.e., in carriers of *APP* duplications) [46, 47].

Unlike *APP* mutations that cluster at the Aβ sequence [43], *PSEN1* and *PSEN2* mutations are found to be widely distributed in these genes (https://www.alzforum.org/alzgene). The majority of them are missense variants and the rest are in-frame deletions/insertions [48–50]. Although *PSEN1* and *PSEN2* are highly similar in terms of their genomic sequences, structures, and function, *PSEN1* mutations appear considerably more pathogenic than *PSEN2* [51]. *PSEN1*-related disease can have an onset as early as the third decade, while symptom onset associated with *PSEN2* mutations shows significant variability and can occur beyond age 65 [51]. Notably, the brain expression of *PSEN2* is ~ 10-fold lower than that of *PSEN1* (https://www.proteinatlas.org/), which could explain more severe disease in *PSEN1* carriers.

### Non-Mendelian EOAD

Only 10–15% of EOAD cases can be explained by known mutations in *PSEN1*, *PSEN2*, and *APP* [33], and a significant portion of unexplained EOAD cases do not show a Mendelian pattern of inheritance, which inherently means that there are other unidentified genetic variations and mechanisms at play. Nevertheless, large-scale genomic studies conducted to date have mostly ignored this form of EOAD. It is likely a complex genetic disease caused by a mix of both rare and common variants, as suggested by a heritability study that assessed the genetic contribution in NM-EOAD cases from 32 US Alzheimer’s Disease Centers to identify the underlying mode of inheritance [3]. This notion is supported by a recent linkage study among multiplex Caribbean Hispanic families loaded for NM-EOAD [52].

Over the past 10 years, genetic and biological studies identified common and rare genetic variants in the Sortilin-related receptor (*SORL1*) gene that are linked to both LOAD and EOAD [53–60]. Functional SORL1 is involved in trafficking of APP protein and can decrease levels of Aβ peptides in a multitude of ways, including Aβ peptides being trafficked to a lysosome for degradation, APP being sent to the Golgi apparatus, and the slowing down of the release of APP from the Golgi [61]. *SORL1* variants could increase the risk of AD by decreasing the affinity for APP at the cell surface [62], thus modifying the role in which SORL1 plays in intracellular trafficking of APP [59, 63]. Recent meta-analyses and genome-wide association studies observed a ~ 20–90% increase in risk of LOAD [54, 64], and a five-fold increased risk of EOAD associated with rare *SORL1* variants [55, 56], which is comparable to the effect size observed in *APOE-e4* carriers [55, 56, 65]. A study that used the Exome Aggregation Consortium (ExAC) database (containing genetic variation of 60,706 human samples [66]) classified the pathogenicity of *SORL1* variants and found that pathogenic *SORL1* variants can increase AD risk by 12-fold as well as cause an earlier age of onset (58.6 ± 5.2 years) for SORL1 carriers [67]. Moreover, it was also found that protein-truncating variants of *SORL1* are highly penetrant, which supports earlier findings that the loss of single copy of *SORL1* is associated with AD [56]. Supporting the notion that these rare variants might explain part of the missing EOAD heritability, these rare protein-truncating *SORL1* variants were observed exclusive in AD cases and absent in the ExAC database [67].

In addition, triggering receptor expressed on myeloid cells 2 (TREM2) has recently been reported to have a significant association with risk of EOAD. Recently, an exome-wide significant association between Arg47His *TREM2* variant and EOAD risk was reported by study of the Alzheimer Disease Exome Sequencing-France dataset consisting of 927 LOAD cases, 852 EOAD cases, and 1273 controls [68]. Principal function of TREM2 is an intracellular adaptor for DNAX-activating protein of 12 kDa (DAP12), as well as a modulator of myeloid cell number, proliferation, and survival [68, 69]. Although the data is inconsistent, rare variants of TREM2 have been associated with Aβ deposition, Aβ uptake in microglia [70–72], increased tau in CSF, and the APOE pathway [69, 73]. Together, these data suggest a strong connection to EOAD neuropathology, as well as both amyloid and tau hypotheses of AD [74].

Both genetic and environmental modifiers of AD risk could act through changes at CpG sites, which could affect DNA methylation (DNAm) levels. Similar to genetic mutations, changes in DNAm could lead to increased or decreased gene expression. Notably, CpGs are the most mutable sites in the genome, since methylated-cytosine can spontaneously change to thymine (C > T transition) [66]. Hence, combined genetic and DNAm studies are important for searching of disease modifiers [75].

Intriguingly, DNAm levels at some CpGs are age-related. The joint assessment of selected age-related CpGs is used in 15 reported DNAm clocks [76]. Each DNAm clock could reflect different aspects of biological aging, the pace of which could vary between different people, in contrast to the steady pace of chronological age. It is critical to investigate the expression patterns of genes containing age-related CpGs. Notably, among 1633 genes contributing to different DNAm clocks, 32 genes involved in the amyloid-β pathway, including *PSEN1* and *BACE1*, which encode γ-secretase and β-secretase, respectively [76].

Studies of DNAm clocks are very relevant to age-related disorders, such as AD. A reliable measure of biological age could be a factor in predicting AD onset in pre-symptomatic mutation carriers. The Horvath’s DNAm clock, which assesses ~ 350 CpGs, outperforms other clocks based on its multi-tissue applicability, including blood and brain [77]. DNAm-age acceleration (a discrepancy between DNAm-age and chronological age) was associated with several neurodegenerative disorders [78–81], including AD [82]. Importantly, DNAm-age acceleration was correlated with levels of Aβ-amyloid pathology in AD brains [83]. However, there is only one study of DNAm-age in familial AD. Recently, we investigated a family with identical triplets affected by LOAD in their late 70s, while one of the offspring developed EOAD at age 50. DNAm-age of the triplets was 6–10 years younger than chronological age. In contrast, DNAm-age was 9 years older in the offspring with EOAD, suggesting accelerated aging [84]. However, it is not clear if acceleration of DNAm-age causes aging or reacts to aging, which could be addressed in future longitudinal studies of DNAm clocks.

## Biofluid and Brain Imaging Biomarkers of EOAD

To support the clinical diagnosis of EOAD in the clinical setting, identifying biochemical changes in CSF, blood or brain imaging are used as diagnostic tools as they can reflect the pathological changes associated with neurodegenerative diseases [85].

### CSF Biomarkers

The most common biomarkers assessed to reflect AD pathology in biofluids are Aβ40, Aβ42, total tau (T-tau), p-tau, and neurofilament light (NFL), an intraneuronal protein and constituent of the axonal cytoskeleton reflecting neuronal degeneration [85]. In CSF, p-tau is measured in order to represent the presence of tau pathology and NFT, whereas T-tau reflects neuronal injury or neurodegeneration [86], although there is evidence that CSF tau might rather represent an increase of neuronal secretion of tau in response to Aβ pathology [87]. The Aβ42 species of Aβ peptides, which is released into CSF [88], is the earliest to accumulate and aggregate into both diffuse and core amyloid plaques [89]. The formation of these hydrophobic plaques consequently lowers Aβ42 that is available to be secreted, causing lower concentration levels of Aβ42 in CSF [85]. Based on the DIAN study, which—as described above—characterizes risk factors, pathophysiology, and biomarker changes across the disease course in carriers of autosomal dominant variants in the *APP*, *PSEN1*, or *PSEN2* genes*,* Aβ is the earliest detectable change to be observed in the neurodegenerative cascade 25 years before estimated age of onset [90]. Fibrillar amyloid demonstrated an annual mean percent change of 6.1%, while there was a decreased rate of change of − 8.8% of CSF Aβ42 that slowed near the estimated age of onset [90]. Decline in precuneus metabolism measured by FDG PET took place 17 years before the estimated age of onset, followed by a decline in cognition and accelerated hippocampal atrophy taking place between 2 and 3 years before the estimated age of onset [90]. CSF p-tau is also seen to decline 3 years before the estimated age of onset in these longitudinal analyses, in contrast to previous observations from cross-sectional analyses that found that CSF tau and p-tau levels are significantly higher 14 and 11 years before estimated age of onset, respectively [90]. Various other studies have confirmed that the decrease of CSF Aβ42 and an increase of CSF tau and p-tau are diagnostically significant in AD dementia cases. A meta-analysis completed on 231 studies found a mean fold change of 0.56 (CSF Aβ42), 2.54 (CSF T-tau), and 1.88 (CSF P-tau) in AD patients, respectively, which shows a stronger association of CSF T-tau and CSF P-tau biomarkers to AD than CSF Aβ42 [91]. In contrast to this M-EOAD, there is a significant lack of studies assessing CSF biomarkers in NM-EOAD and potential differing patterns from autosomal dominant EOAD and LOAD. Clarifying longitudinal changes in these biomarkers from early adulthood and across the disease course will be critical to disentangle the molecular distinction of NM-EOAD from M-EOAD and LOAD and improve the use of CSF-based biomarkers for screening and diagnosis in the clinical setting.

### Plasma Biomarkers

Although measuring changes in CSF is advantageous because of its contact with the extracellular space of the brain, over the past two decades, significant efforts have been made to develop reliable, sensitive, and specific biomarkers of AD measured from blood as they are less invasive and significantly easier to obtain in both clinical and research settings [85]. While the close contact of CSF with the brain results in relatively high levels of molecules associated with brain disease, much lower amounts exist in the bloodstream. Additionally, plasma Aβ can originate from other organs and tissues as well as be broken down by proteases, metabolized in the liver, or cleared by the kidneys [85, 92], all of which can influence the concentration of Aβ in the bloodstream. However, recent technological advancements in ultrasensitive immunoassays (such as the single-molecule array (Simoa technology) and mass spectrometry to assess plasma levels of molecules are providing promising results of the usefulness of these technologies for diagnosis, prognosis, and monitoring of progression of AD [93, 94].

Recent studies demonstrated that in particular the Aβ42/40 ratio measured by ultrasensitive assays provides a sensitive assessment of amyloid burden that correlates both with the Aβ42/40 ratio in CSF and amyloid PET positivity in both M-EOAD and LOAD [95–98]. In addition, several studies have shown that NFL has diagnostic accuracy for AD similar to that of CSF NFL and increases over time as brain atrophy increases and cognition declines [99, 100]. Studies in individuals with M-EOAD including the DIAN study further indicate that, similar to CSF, plasma NFL is elevated more than a decade prior to symptom onset, suggesting that it can serve as a sensitive diagnostic screening tool to identify individuals at risk [101]. While p-tau181 levels are elevated in AD, show associations with both Aβ and tau PET using agents such as C-PiB (Aβ) and ^18^F-AV-1451(tau) (previously known as T807) [102–104], suggesting greater specificity for AD pathology than other tau species [105], overall the correlation of tau species measured from blood with CSF tau is relatively weak, potentially limiting the usefulness of currently available tau assays for screening, diagnosis, and monitoring of disease progression.

While it is reasonable to postulate that the abovementioned blood-based biomarker changes might behave similarly in NM-EOAD, there is a lack of studies assessing the prognostic and diagnostic validity of these measures in this type of AD.

## Neuroimaging

Neuroimaging techniques done in both clinical and AD research settings include structural magnetic resonance imaging (MRI) and fluorodeoxyglucose (FDG**)-**positron emission tomography (PET), although the latter is often not covered by insurance and thus not performed. Structural MRIs assess the volumetric atrophy seen in AD, while FDG-PET identifies altered glucose metabolism. Current data on M-EOAD continues to parallel the typical presentation of glucose hypometabolism, amyloid deposition, and atrophy that is also seen across many cortical regions (thalamus, putamen, pallidum, hippocampus, caudate, amygdala, and accumbens) in LOAD cases [106]. However, besides the fact that in M-EOAD these changes are seen earlier, they are often more widespread affecting additional areas including the putamen and thalamus [107], and there is often substantial hypometabolism seen first in posterior cingulate/precuneus and lateral parietal regions before it extends to include the frontal and temporal cortices [106]. Data from the DIAN study suggests that carriers of causal mutations exhibit cortical glucose hypometabolism and cortical thinning in the medial and lateral parietal lobes 5–10 years before the estimated age of onset, which suggests that FDG-PET and MRI changes are contemporaneous [106]. Subcortical regions seem to show a differing pattern. While all subcortical gray matter regions exhibited elevated PiB uptake, only the hippocampus showed reduced glucose metabolism [106].

Imaging AD pathology also includes the use of amyloid imaging tracers such as Pittsburgh Compound-B (PiB), as well as three amyloid radiotracers Amyvid™ (florbetapir F18), Neuraceq™ (florbetaben F18) and Vizamyl™ (flutemetamol F18) and one tau ligand (Tauvid™; 18F-flortaucipir) that are now approved by the Food and Drug Administration (FDA) [108].

In line with the morphological and metabolic changes that are seen before the estimated age of onset, elevated PiB levels are observed in almost every cortical region in an evenly dispersed manner almost 15 years before estimated age of onset [106]. However, the pallidum and caudate are exceptions because there is an observed increase of PiB levels but no evidence of metabolic or volumetric changes, which demonstrates that not all regions follow typical biomarker ordering [106]. In company with the elevated levels of PiB, tau-PET imaging demonstrates an increased level of tau tracer binding in the neocortex, and when compared to sporadic LOAD the levels of both agents are higher in M-EOAD [109]. Correlations between the specific component of tauopathy and the observed increase in tau-PET signaling are still unclear.

In line with M-EOAD, NM-EOAD sporadic cases with no family history of AD when compared to LOAD also present with more atrophy [110], robust neocortical glucose hypometabolism [111], and increased tau-PET signaling [112, 113]. Although imaging studies are limited, these findings match the neurodegeneration and tauopathy that are seen in brain autopsies and show a more aggressive disease progression at younger age of onset. These observations are also reflected in analyses of the Alzheimer’s Disease Neuroimaging Initiative (ADNI) dataset (unrelated > 65-year-old subjects) showing more severe changes and disease progression MRI and biofluid-based biomarkers in persons with younger compared to older onset [114].

## Conclusion

While there have been important advances in AD research in the past decades in understanding the mechanisms underlying EOAD, a significant part of its etiology remains unclear, hampering more accurate diagnosis and more effective treatment in the clinical setting. While routine screening using whole-exome sequencing is now feasible in the clinical setting, its interpretation (and associated genetic counseling) is largely limited to known mutations in *APP*, *PSEN1*, *PSEN2, GRN*, and *MPT,* which only account for a small subset of EOAD cases before age 65. Clarifying the genetic, molecular, and clinical distinction of unexplained non-Mendelian EOAD from M-EOAD and LOAD, its pattern of cognitive decline, biomarker, and neuropathological changes over the disease course and the genetic and molecular underpinnings of its observed clinical variability will be critical to improve both diagnostic screening as well as developing more effective preventive and therapeutic targets. To do this, cohorts of NM-EOAD cases with sufficient sample size, deep phenotypic characterization, longitudinal follow-up, comprehensive AD biomarker assessment, and an array of integrative data (i.e., genomic, epigenetic, proteomic, transcriptomic and metabolic data) are needed. Ideally, these datasets would comprise both family-based and case-control cohorts of various ancestral backgrounds to disentangle genetic variation specific to ethnic group, familial aggregation, or sporadic occurrence. Next-generation sequencing data coupled with other multi-omics approaches and extensive bioinformatics in such datasets would further allow to identify allelic effectors of identified loci, molecular underpinnings of disease etiology, progression and variation in age at onset, and the delineation between clinical endophenotypes. Identification of molecular disease signatures and pattern(s) of altered protein expression would allow to discriminate early disease stages, improve disease screening and diagnostics, identify subjects at risk for disease or more severe progression, and facilitate the identification of “druggable” molecular targets and successful repositioning of drugs for clinical application. Application of standardized assessment and analysis protocols comparable to those of the ongoing major studies on M-EOAD and LOAD will be critical to allow for valid comparison of data with these forms of AD.

As described above, the ADNI/DIAN Neuropathology Core has implemented a uniform assessment protocol to evaluate the neuropathological overlap between M-EOAD and LOAD. Similar collections of NM-EOAD brain tissue with standardized neuropathological assessment are needed to comprehensively delineate neuropathological overlap of both these forms with sporadic and familial NM-EOAD. Given that the NM-EOAD is likely heterogeneous, genetic analyses should test a variety of inheritance patters including recessive and polygenic effects.

Finally, there is a critical need for more studies to authenticate the use of ultrasensitive blood-based biomarkers as a screening, diagnostic, and monitoring tool in EOAD subtypes. Current data show that in particular the Aβ42/40 ratio and NFL measured from blood using new technologies show a high degree of specificity and sensitivity to detect AD in individual years before onset of symptoms and correlate with CSF and imaging changes over the disease course. Validation of course and pattern of blood-based biomarkers in EOAD subtypes, ideally informed by genetic background, would be highly valuable for both clinical and research settings.

In-depth characterization of the clinical, genetic, and neuropathological distinction of unexplained EOAD from M-EOAD and LOAD is critical to fully understand the etiology of AD, characterize determinants and modulators of risk and age at onset, refine prevalence and incidence, and develop more effective targets for screening, prevention, and treatment.

## Data Availability

N/A.
